# Estuarine dispersal of an invasive Holarctic predator (*Esox lucius*) confirmed in North America

**DOI:** 10.1371/journal.pone.0315320

**Published:** 2024-12-27

**Authors:** Matthew J. Wooller, Parker Bradley, Karen J. Spaleta, Robert L. Massengill, Kristine Dunker, Peter A. H. Westley

**Affiliations:** 1 Alaska Stable Isotope Facility, University of Alaska Fairbanks, Fairbanks, Alaska, United States of America; 2 College of Fisheries and Ocean Sciences, University of Alaska Fairbanks, Fairbanks, Alaska, United States of America; 3 Sport Fish Division, Alaska Department of Fish and Game, Palmer, Alaska, United States of America; 4 Sport Fish Division, Alaska Department of Fish and Game, Anchorage, Alaska, United States of America; University of Maryland Center for Environmental Science, UNITED STATES OF AMERICA

## Abstract

The capacity for a non-native species to become invasive largely hinges on existing dispersal capacity or adaptation of dispersal in new environments. Here we provide early evidence that invasive Northern Pike (*Esox lucius*), a Holarctic freshwater top predator, illegally introduced in the late 1950s into Southcentral Alaska, are now dispersing through estuarine corridors. This finding represents the first known documentation of estuary use and dispersal by Northern Pike in North America, exacerbating conservation concerns for already depressed populations of culturally and economically important species such as salmonids. To reconstruct the migratory pathway of individual Northern Pike captured at locations known to be recently invaded, we analyzed the strontium isotope composition (^87^Sr/^86^Sr) in otoliths. In Vogel Lake, where Northern Pike were first observed in 2019, the smallest (youngest) Northern Pike collected had relatively constant ^87^Sr/^86^Sr values consistent with ^87^Sr/^86^Sr values of freshwater samples from the region and indicating a wholly freshwater existence. However, the largest Northern Pike (95.5 cm) in Vogel Lake had isotopic signatures indicating its early life had been in an estuarine habitat before moving into Vogel Lake through a short 4.8 km creek connecting it to the ocean. We subsequently analyzed otoliths from two other Northern Pike, from two additional separate locations in Southcentral Alaska, revealing signatures consistent with colonization through an estuarine corridor. It is unclear whether estuarine dispersal ability has evolved *de novo* in these Northern Pike populations or was retained by plasticity. Regardless, this early evidence is of considerable concern in Alaska and other regions of North America confronting Northern Pike introductions and underscores the urgency to monitor connected freshwater systems most vulnerable to invasion via adjoining estuarine habitats.

## Introduction

Dispersal is a fundamental biological phenomenon that shapes population ecology and evolution and mediates the spread of non-native species following introduction [1, 2]. Across taxa, dispersal capacity is positively associated with the probability of invasiveness [3], which can be influenced by climatic conditions such as changing patterns of freshwater flow [4] and, in some species, can evolve following introduction [5]. For many species, dispersal limitation is in part imposed by physiological constraints to gradients in temperature, elevation, or salinity [6], and understanding the factors that act as barriers and bridges to dispersal is a key question in invasion biology. Good candidate species for providing insight come from those that disperse between ecosystems, such as freshwater and marine domains.

Northern Pike (*Esox lucius*) have a freshwater and native Holarctic circumpolar distribution, including northern Europe, Asia, and North America, and are generally found above 40° latitude [7, 8]. Although Northern Pike have rather confined home ranges with relatively short dispersal events, illegal anthropogenic introductions of Northern Pike have expanded their range in several countries, including throughout the American West region of North America [9]. The historic distribution of Northern Pike in Alaska is largely the result of geologic barriers present during the Late Pleistocene when the majority of southern Alaska was glaciated [10]. While Northern Pike inhabited the unglaciated parts of interior Alaska thousands of years ago [11], freshwater fish assemblages in drainages in Southcentral Alaska, defined as the region south and east of the Alaska Mountain Range, developed in the absence of this aquatic apex piscivore [12, 13]. Local residents have anecdotally reported that an angler transported Northern Pike in the late 1950s from the Minto Flats in Interior Alaska to a lake in the Susitna River basin. Seasonal flooding events, other illegal introductions, and pike movements have resulted in the establishment of Northern Pike in >150 lakes and rivers in the vicinity of Anchorage as well as several drainages in Southcentral Alaska, including the Knik Arm drainage, Susitna drainage, northern Kenai Peninsula and west Cook Inlet drainages [9, 14]. Northern Pike, as a top predator, can prey on juvenile salmon and have the potential to devastate economically important fisheries as well as native fish populations [14–17]. Over the last decade, significant resources from multiple organizations have also been allocated toward invasive Northern Pike eradication, suppression, and monitoring [9, 18]. A management plan for mitigating invasive Northern Pike in Southcentral Alaska was published in 2022 [18]. This management plan was formulated in partnership with the Alaska Department of Fish and Game (ADF&G), federal agencies, non-governmental organizations, universities, Alaska Native organizations and local communities and has highlighted several objectives for the next decade. These objectives include minimizing intentional invasive Northern Pike introductions and reintroductions; implementing scientifically sound management options to detect, eradicate, contain, or suppress invasive pike populations and conducting research to fill knowledge gaps that could improve management of invasive pike [18]. One of the largest knowledge gaps for the region concerns understanding the degree of mobility of Northern Pike.

**Fig 1:**
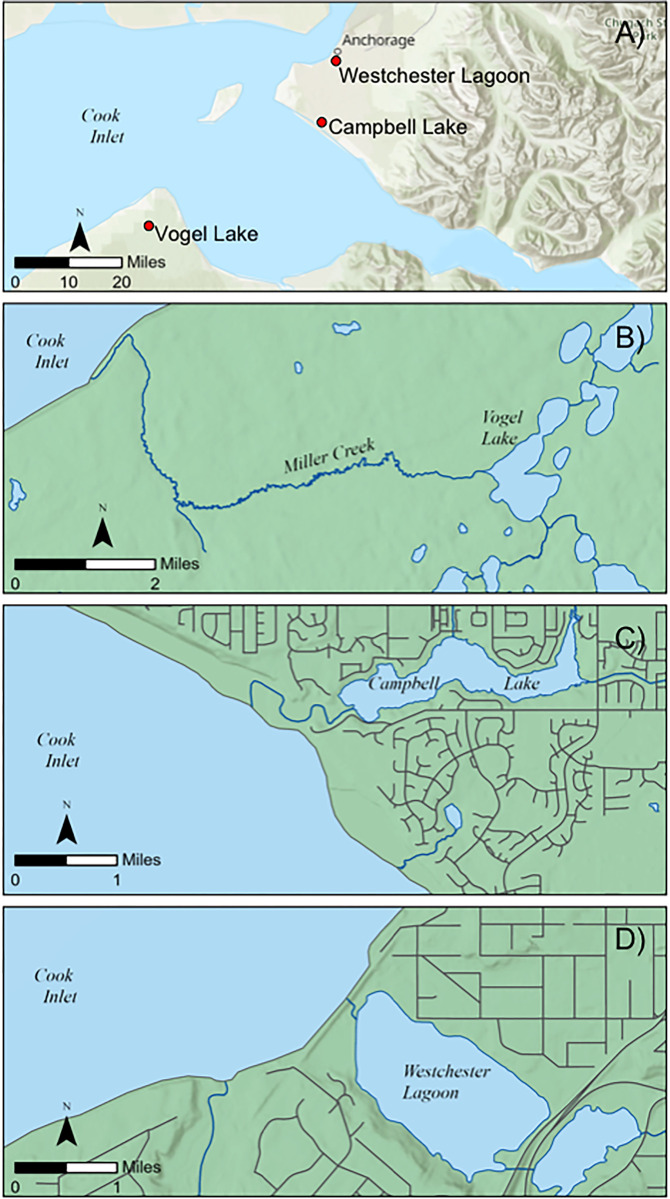
Study lake locations in Southcentral Alaska (A), including Vogel Lake (B), Campbell Lake (C) and Westchester Lagoon (D).

Although Northern Pike are considered a freshwater fish, they have broad physiochemical tolerances and can withstand a wide range of water quality conditions, including living in estuaries between 10–12 ppt [19] and are present in the Baltic and Caspian Seas [20–22]. A recent investigation into salinity tolerances of Northern Pike in Southcentral Alaska revealed their ability to survive at least 96 hours in 14–21 ppt [23]. Their movement patterns can also be variable, with some populations aggregating in estuaries [24]. In Southcentral Alaska, commercial salmon set netters have occasionally reported catching Northern Pike in the estuarine habitats of Cook Inlet [ADF&G Unpublished Data]. In May 2019, Northern Pike were discovered for the first time on the northern tip of the Kenai Peninsula in Vogel Lake, in the Miller Creek drainage. New populations of Northern Pike were also discovered in Campbell Lake and West Chester Lagoon in Anchorage in 2022 (Fig 1).

ADF&G’s management activities of Northern Pike thus far have included the collection, preservation, and archiving of thousands of otoliths (ear stones) from captured Northern Pike. Otoliths, which are part of a fish’s inner ear, are tree-ring-like structures that preserve a lifetime chemical record of the locations a fish has lived. Otoliths are used for hearing and balance in teleost fishes and grow incrementally, forming concentric layers primarily composed of calcium carbonate. Once laid down, each layer is metabolically inert and retains a signature of the location from which it was laid down. Isotope techniques have previously been used to reveal the diet, biogeography, and movement ecology of Northern Pike [11, 25–27], and strontium (Sr) isotope ratios (^87^Sr/^86^Sr) can be measured on fish otoliths to reveal movement behavior and dispersal strategies [28–30]. ^87^Sr/^86^Sr in otoliths are an especially effective way to understand movement ecology and habitat use of fishes [28–30], including Northern Pike [27], because different aquatic habitats can have very different ratios of ^87^Sr to ^86^Sr. Concurrent measurement of ^88^Sr (presented here as a voltage (V) measured on the detector) yields a semi-quantitative proxy for the overall concentration of total Sr, which can further help discern habitat changes. ^87^Sr/^86^Sr differences are driven by differences in habitat lithology, geologic age, chemical composition, and weathering rates of surficial geology [31, 32]. Fish take up dissolved Sr from water through their gills, which in turn substitutes for some calcium during mineralization of the incremental otolith layers. In this way, otoliths provide a continuous record of Sr that directly reflects habitats used during the lifetime of a fish. Here we seek to understand where Northern Pike have come from by analyzing the ^87^Sr/^86^Sr and ^88^Sr in the otoliths of several Northern Pike captured in Vogel Lake, Campbell Lake and Westchester Lagoon, Southcentral Alaska. In particular, we test whether Northern Pike from these systems show signals consistent with having been in estuarine habitats prior to their capture in freshwater.

## Materials and methods

ADF&G staff collected sagittal otoliths from Northern Pike captured at Vogel Lake, Campbell Lake, and the Westchester Lagoon (Fig 1). Northern Pike were captured using monofilament gillnets, with methods and net specifications following a standard operating procedure outlined in the Technical Guidance and Management Plan for Invasive Northern Pike in Southcentral Alaska [9]. ADF&G do not work under collection permits, unless fish are being moved, which was not the case here. For ADF&G, fish collections are considered part of standard operations. Gillnets were used to capture Northern Pike from our study locations. Nets were 37 m in length by 2 m in depth and composed of 6 panels of differing mesh sizes ordered in size along the length: 19 mm (0.75 in), 25 mm (1.0 in), 31 mm (1.25 in), 38 mm (1.5 in), 44 mm (1.75 in), and 51 mm (2 in). All nets were made of monofilament with a 12.7 mm (0.5 in) foam float line and 22.6 kg (50 lb) lead line.

Sampling took place in littoral habitats where the lead line sits on the bottom and the float line is on the water surface. Initial sampling in May 2019 at Vogel Lake (Fig 1B) produced one large, old female (length 95.5 cm, age 6, born in 2013), and several other smaller and younger individuals (length 36.1 to 68.0 cm, age 3 and younger, born 2016 and later) (Table 1). Sampling at Vogel Lake also occurred in July 2019, September 2019, May 2020, July 2021, September 2021, and October 2021. Aging was conducted using cleithra following previously published protocols [33]. All subsequent fish captured in the later sampling events were age ~0–3. Otoliths from these individuals were sent to the Alaska Stable Isotope Facility (ASIF) at the University of Alaska Fairbanks (UAF’s) Troth Yeddha’ campus for preparation and analysis.

**Table 1:** Location, size and sex (where known) of individuals analyzed in this study.

Site location	Fork Length (cm)	Age	Sex
Vogel Lake	95.5	6	Female
Vogel Lake	68.0	3	Male
Vogel Lake	58.5	2	Female
Vogel Lake	58.6	2	Female
Vogel Lake	55.0	2	Female
Vogel Lake	55.2	2	Male
Vogel Lake	36.1	1	Female
Campbell Lake	68.7	3	Female
Westchester Lagoon	64.0	3	Female

In 2022, Northern Pike were also discovered in two new locations in Anchorage, Campbell Lake and Westchester Lagoon, both of which have very short connections to Cook Inlet (Fig 1C and 1D). Campbell Lake and Westchester Lagoon are within two primary drainages, Campbell Creek and Chester Creek, respectively, in the Anchorage municipality, and both support anadromous species. Both lake systems are created by outlet dams that allow fish passage. In Campbell Lake, 24 Northern Pike were captured in 2022, mostly of 1- and 2-year-olds. However, one 3-year-old female was also captured. In Westchester Lagoon, 5 Northern Pike were captured in 2022, all of which were age 0 and age 1, except one age-3 female. The otoliths from these 3-year-old fish were sent to ASIF for preparation and analysis.

**Fig 2:**
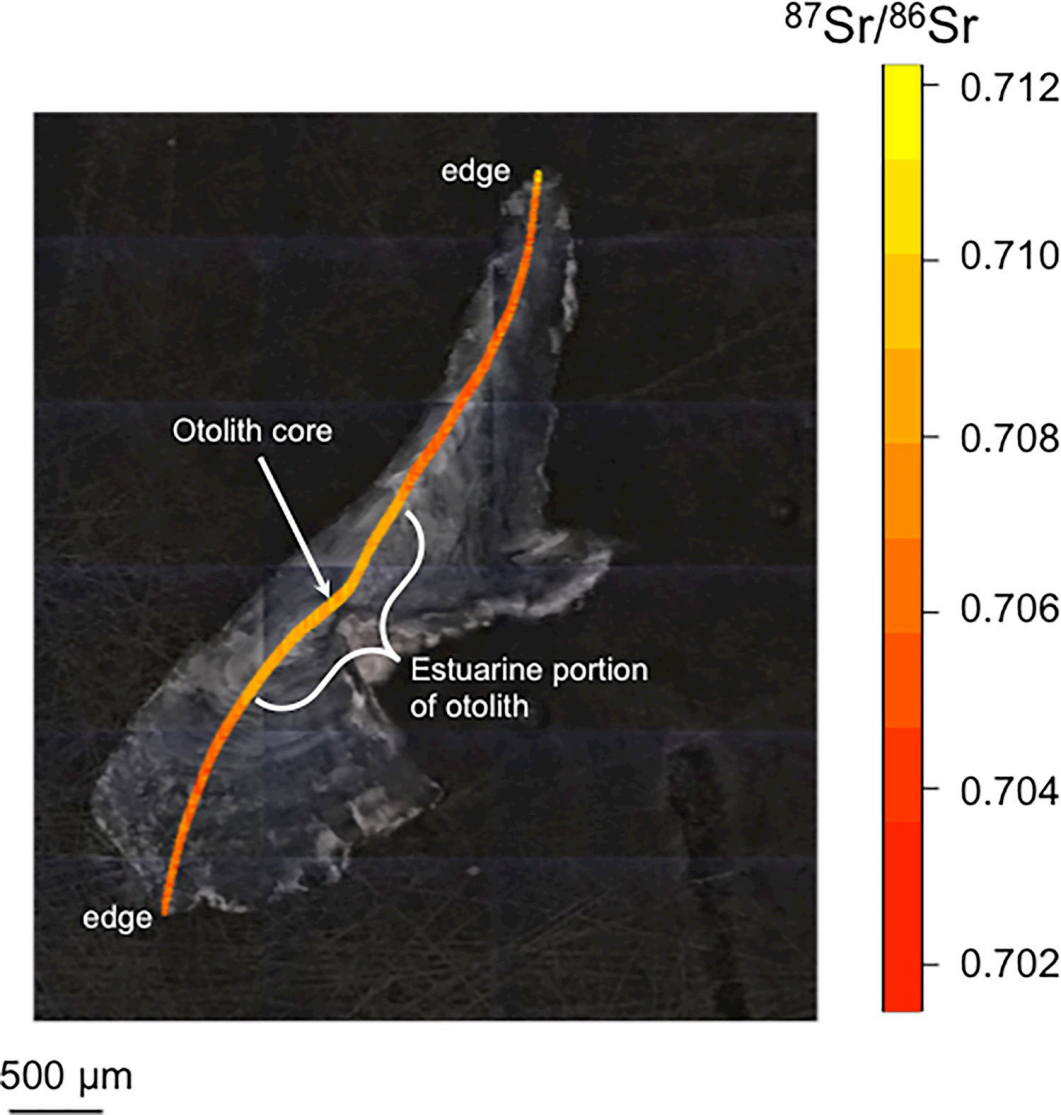
An annotated example of a Northern Pike otolith.

Our otolith preparation and analysis followed previously published protocols [34]. In brief, we embedded otoliths in molds using Buehler Epothin 2 epoxy. Otoliths (n = 9) were thin- sectioned in the transverse plane and mounted on individual coverslips using Crystalbond^TM^ 509, and sequentially wet polished with 800 and 4000 grit SiC polishing paper. A final polish was achieved with a 3-μm alumina slurry on glass. We then used an Analyte G2 Excimer 193-nm Laser Ablation System (LA; Teledyne Photon Machines, Bozeman, USA) with a Helex cell coupled to a Neptune Plus^TM^ multi-collector inductively coupled plasma mass spectrometer (MC-ICP-MS; Thermo Scientific^TM^, Bremen, Germany) for strontium isotope analyses at UAF’s ASIF. We selected an ablation path perpendicular to annuli across each otolith (Fig 2).

For isotope analysis, the laser was set to 35 μm beam diameter, a pulse rate of 10 Hz, a scan speed of 5 μm/s, and a laser fluence of 6.64 J/cm^2^. The laser stream was mixed with the output of an Aridus II^TM^ membrane desolvation system via a mixing chamber immediately prior to the plasma inlet. We used a National Institute of Standards and Technology (NIST) solution of ~13 ng g^-1^ SRM 987 (SrCO_3_ Sr isotopic standard) and ~20 ng g^-1^ Zr standard to optimize the instrument and also aspirated via the Aridus II^TM^ every 30 minutes for IIF correction via bracketing from standard to sample acquisition. While we ablated samples, the Aridus II^TM^ aspirated a 2% solution of HNO_3_ that was double distilled via sub-boiling distillation (Savillex DST-1000) from trace metal grade concentrated HNO_3_. We then used American Society for Testing and Materials (ASTM) Type I water from a Milli-Q1 IQ 7000 system, which had been further purified via a double sub-boiling distillation (Savillex DST-1000) for dilution.

Our ablation data was processed following previously published procedures [35]. In brief, we blank-corrected data by subtracting the mean gas blank values obtained during the 30-second laser-warmup period prior to each ablation for all measured isotopes. We calculated calcium (Ca) dimer (CaCa) and Ca argide (CaAr) corrections based on the blank corrected signals at mass to charge number (m/z = 82 and 83) using the natural abundances of Ca and argon (Ar) and applied to the blank corrected signals at m/z 84, 85, 86, 87, and 88. We calculated rubidium (Rb) interference correction on m/z 87 using Russell’s law via mass 85 and applied via peak stripping in which the fractionation of Rb was assumed to be equal to Sr due to the low levels of Rb naturally occurring in sample materials like otoliths. We corrected the ^87^Sr/^86^Sr via standard/sample bracketing of the NIST SRM 987 solution. Instrumental isotopic fractionation (IIF) was corrected externally, also using the NIST SRM 987 solution. Otolith ^87^Sr/^86^Sr data post-processing followed previously published protocols [28]. We individually inspected strontium data from otolith laser ablation for data quality, trimmed, and adjusted accordingly. Our laser ablation analysis began at one edge of each otolith and proceeded across the entire otolith, passing through the otolith’s core (Fig 2). This means that the left and right edges of the data profile from each of the otoliths represents the end of the fish’s life, while the center of the profile represents when the fish was born (the otolith core). The data subsequently is typically very similar either side of the core. Finally, we compared the otolith ^87^Sr/^86^Sr values with a suite of ^87^Sr/^86^Sr values we have previously generated for the region [36]. This included collecting water samples from various lakes, rivers and estuarine locations throughout the region to measure their ^87^Sr/^86^Sr values, including locations in the vicinity of Anchorage, where both Westchester and Campbell lakes are located, as well on the Kenai Peninsula, where Vogel Lake is located. Marine habitats generally have higher concentrations of Sr (therein higher measured ^88^Sr) and relatively homogenous ^87^Sr/^86^Sr values (global marine value = ~0.70918) compared to freshwater habitats [36]. These relatively high ^87^Sr/^86^Sr values contrast with the average ^87^Sr/^86^Sr for the Kenai Peninsula (0.7063) and the relatively low values for locations in the vicinity of Anchorage [36].

## Results

**Fig 3:**
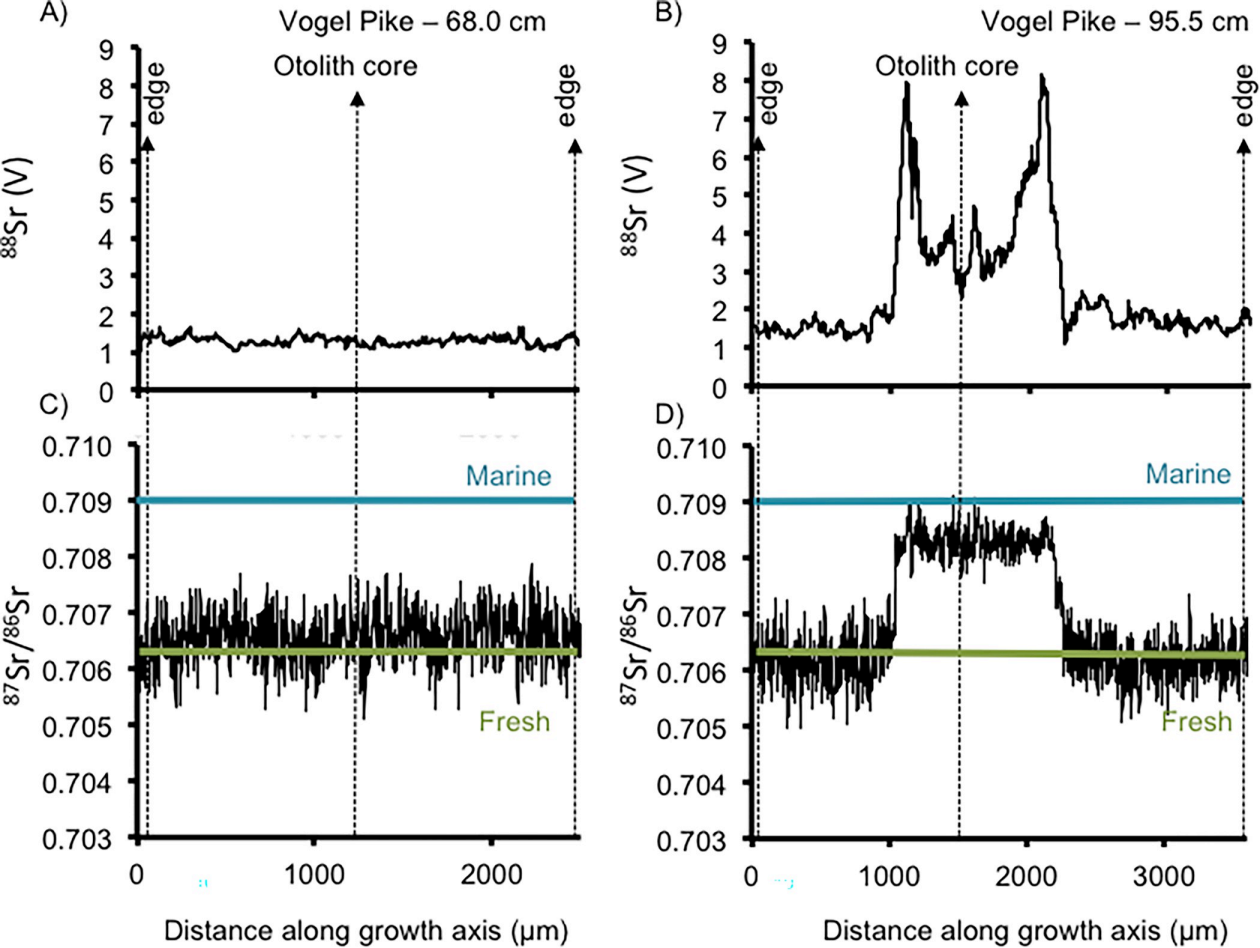
Strontium ‘concentration’ as a voltage (^88^Sr) (A,B) and strontium isotope ratios (^87^Sr/^86^Sr) (C,D) from otoliths of two northern pike (Vogel Pike #2–68.0 cm (age 3) and Vogel Pike—95.5 cm) removed from Vogel Lake on the Kenai Peninsula.

We detected signals of both freshwater residency and usage of brackish estuarine habitats, but the detection varied by age and size of the individual. For example, the 6 smaller individuals of Northern Pike analyzed from Vogel Lake exhibited minimal ^87^Sr/^86^Sr variation during their lifetimes (an example of one of these Northern Pike is shown in Fig 3A and 3C and others are shown in the S1–S5 Figs in S1 File). Their average ^87^Sr/^86^Sr values were consistent with the average ^87^Sr/^86^Sr for the Kenai Peninsula (0.7063) [36]. The ^88^Sr voltage (V) signals for all these smaller fish were <4.5 (ranging from ~1.0 to 4.5). In contrast, the ^87^Sr/^86^Sr and ^88^Sr V data from the largest (95.5 cm long) Northern Pike captured from Vogel Lake had ^87^Sr/^86^Sr values (up to 0.709) from the early stage of its lifetime (i.e., towards the center of the otolith) that were notably higher (i.e., relative to analytical precision) than the Vogel Lake region (i.e., ~0.7063) (Fig 3D). These higher values are indicative of estuarine values. The early period of this Northern Pike’s life also showed much higher ^88^Sr V data (up to ~8 V) than the entire range of the smaller Northern Pike. These higher values are also consistent with estuarine conditions where strontium is much more abundant.

**Fig 4:**
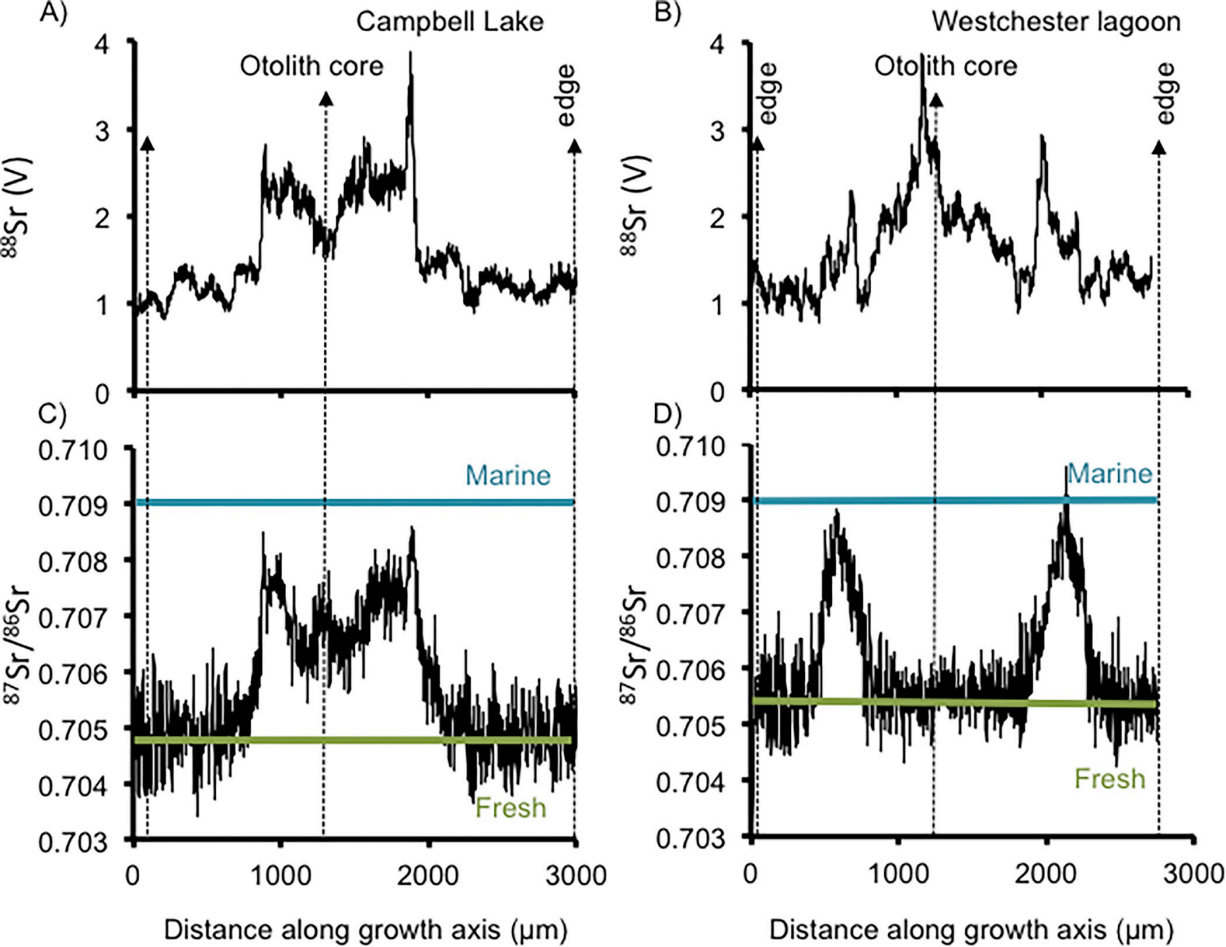
Strontium ‘concentration’ as a voltage (^88^Sr) (A,B) and strontium isotope ratios (Sr^87^/Sr^86^) (C, D) from otoliths of two northern pike removed from Campbell Lake and Westchester Lagoon.

The Northern Pike analyzed from Campbell Lake also seems to have spent time in an estuarine environment. Although the ^88^Sr V in the central part of this Northern Pike’s otolith (Fig 4A and 4C) does not approach the higher values seen in the large Northern Pike from Vogel Lake, they are elevated in the central part of the otolith relative to the edges (Fig 4A). These higher ^88^Sr V values also correspond with elevated ^87^Sr/^86^Sr values that also reach estuarine-like values (Fig 4C). Additionally, there is a spike in ^87^Sr/^86^Sr values between the core and the edge of the otolith where the values are consistent with Cook Inlet values, suggesting a brief exposure to the estuarine environment immediately before being exposed to (entering) Campbell Lake.

Results of the ^87^Sr/^86^Sr analysis of the individual from Westchester Lagoon indicate ^87^Sr/^86^Sr values of roughly 0.7055 in the core and the edge. However, there is a significant spike in ratios that are consistent with Cook Inlet brackish water (Fig 4D), which also corresponds with an increase in ^88^Sr V (Fig 4B).

## Discussion

The spread of invasive species, especially those of top predators, is a key conservation concern requiring early detection and rapid responses [37]. Here, we show early evidence of Northern Pike captured from three separate locations in Southcentral Alaska that indicate a previously undocumented behavior for Northern Pike in North America. Northern Pike are presumed to be an obligate freshwater species in North America, but now appear capable of dispersing through brackish estuarine habitats. This is a behavior that has also been documented for some other fish species (e.g., Blue Catfish) that have been introduced into freshwater habitats but have then expanded into higher salinities [38]. The Sr data from all of the smallest Northern Pike from Vogel Lake are consistent with these fish having resided and grown in the Vogel Lake watershed with no evidence of any movement from this location. However, the results from the largest female caught at Vogel Lake not only indicate that it originated from another location outside of the Miller Creek drainage (Fig 3B and 3D), but it also passed through an estuarine/brackish environment first. Although it is difficult to pinpoint the origin of this fish as ^87^Sr/^86^Sr data for every single drainage in the region is not available, we can confidently conclude it did not come from the Miller Creek drainage based on the known ^87^Sr/^86^Sr values for this area (0.7063) [36]. The core ^87^Sr/^86^Sr value in this Northern Pike was around 0.7084. Additionally, the sudden decrease in ^87^Sr/^86^Sr values and ^88^Sr V to values that reflect Miller Creek water immediately following the estuarine signature suggest it moved directly from the estuarine environment into the Miller Creek drainage. Vogel Lake and Miller Creek drainage are remote and very inaccessible to most people and we therefore speculate that human-mediated pathway for this fish is unlikely. Based on ages of fish captured in Vogel Lake, the movement of the founding fish likely occurred in 2014 or 2015. This fish would then have been able to spawn in the spring of 2016 at the age of three, producing the population in Vogel Lake. Northern Pike are broadcast spawners so establishment would have required at least one adult male Northern Pike to be present in the system during that time, which presumably also must have traveled up Miller Creek and either died before sampling or was never captured. These types of inferences would benefit from genetic analyses and parental assignments. The population of Northern Pike in the Miller Creek drainage was eradicated in the fall of 2021 [18] to prevent this population from expanding its range on the Kenai National Wildlife Refuge and northern Kenai Peninsula. Since then, a fish weir in Miller Creek has been in operation to prevent the potential return of Northern Pike to this drainage via Cook Inlet.

Otolith data collected from a Northern Pike from Campbell Lake indicate this fish also moved between different habitats that included marine/estuarine locations. However, this female was likely not the founding fish in Campbell Lake. Northern Pike spawn in the spring, generally beginning under ice cover in April and continuing through the end of May, though this is site-specific. Females become mature at 2–3 years of age [39]. The female analyzed from Campbell Lake would have needed to have been age-4 to have produced the other Northern Pike in the system that are age-2. This suggests besides obviously another male, another female would have had to have been in the system to produce this population of younger fish (origin or pathway unknown). In this scenario, the Campbell Lake female Northern Pike briefly moved into Cook Inlet, and then entered Campbell Lake where she was captured in ADF&G’s gillnets. Therefore, results from this fish suggest migration through Cook Inlet is a viable and likely pathway into this system.

The otolith data from the Northern Pike captured from Westchester Lagoon suggests two possible scenarios: 1. This Northern Pike was born in the Chester Lagoon system, made a brief movement out of the lagoon and into Cook Inlet, and then returned to Westchester Lagoon. 2. This Northern Pike was born in another system with a similar ^87^Sr/^86^Sr value as the Westchester Lagoon system, moved into Cook Inlet, and then moved into Westchester Lagoon. The ^88^Sr V signal from the Northern Pike’s otolith being slightly higher in the central part of the otolith than the edge, indicates that this Northern Pike is likely of a different origin than the Westchester Lagoon system. The age of this Northern Pike (age-3) is consistent with being a potential founder fish, as the only other captured Northern Pike from this location were age 0 and 1.

Whereas Northern Pike have been observed in estuarine environments in Europe [20–22], the data here provide the first empirical evidence indicating that Northern Pike can use estuarine pathways to aid dispersal in Alaska, representing a new invasion pathway in North America. We acknowledge that in some fish species, consumption of marine-derived nutrients delivered to freshwater habitats, such as eggs from anadromous fish can produce marine-like signatures in otoliths [40], although this prior research has focused on studying the strontium calcium ratios in otoliths rather than focusing on the strontium isotopic compositions (i.e., ^87^Sr/^86^Sr) of otoliths. However, given such short river access to estuarine habitats of the Cook Inlet (Fig 1) and that the Northern Pike we analyzed represent the first appearance at the sites we report on, we consider that the most parsimonious explanation of our otolith findings is that some Northern Pike spent time moving through estuarine locations. These inferences are also consistent with some anecdotal reports from local fishermen of having caught Northern Pike in commercial setnets set in estuarine locations [18].

We acknowledge that our sample size is small and that moving forward this type of investigation would benefit from analyses of more individuals from sites throughout the watershed. However, given the novelty of our findings to North America and the potential implications for wildlife management and broader ecosystem health, there is now a need to better understand the prevalence of this behavior as well as the dispersal and movement of Northern Pike in Southcentral Alaska [18]. Future research should be dedicated to further analyses of Northern Pike otoliths from the region. Otoliths (n = 3000+) have already been collected from Northern Pike captured by our collaborators over the last 5 years during Northern Pike eradication and suppression efforts in Southcentral Alaska. These otoliths could form a valuable basis for a large-scale, spatiotemporal assessment of Northern Pike movement patterns in the watershed. Some additional critical research would be documenting salinity values throughout Cook Inlet, coupled with further investigations into the tolerances of Northern Pike to these values. This would help determine the freshwater drainages most at risk of Northern Pike invasion via estuarine pathways in Alaska, and be insightful for other locations where invasive Northern Pike populations are present in coastal drainages [14]. Future research efforts could also be dedicated to enhancing the spatial resolution of the strontium isoscape (map) for Southcentral Alaska by analyzing more water samples. We have also initiated genetic analyses of the individuals we have presented here alongside analyses of other comparative Northern Pike from Alaska, to investigate the potential for adaptation of Northern Pike to cope with more saline habitats. Isotopic data coupled with genetic data from the same individuals will provide a holistic perspective into life history, biogeography and population dynamics [7, 8]. Our efforts here are part of a long-term and comprehensive effort being led by ADF&G to illuminate and limit the impact of Northern Pike invasion in Southcentral Alaska. This is including genetic investigations of population structure, under ice winter monitoring using underwater remotely operated vehicles, under ice set nets to extend the survey season for monitoring sites, widespread eradication efforts, and salinity trials to test the tolerance of Northern Pike from Southcentral Alaska to a range of habitat waters of differing salinity levels. A controlled experiment is currently being designed and led by ADF&G to also expose Northern Pike to a range of salinities and strontium isotope compositions to examine how these conditions are reflected in otoliths. This integrated pest management approach to control invasive Northern Pike in Southcentral Alaska, which includes strontium isotope analyses of otoliths from field sites, formed the basis for a recent, comprehensive management plan [18]. This management plan was led by ADF&G in close collaboration and partnership with other agencies, academics and community partners and is forming the basis for formulating future and ongoing management and research initiatives.

## Supporting information

S1 File S1–S5 Figs. (PDF)

S1 Table Strontium isotope data of the pike presented. (XLSX)
