# Supplementary material for: Estuarine dispersal of an invasive Holarctic predator (Esox lucius) confirmed in North America
Source: PLoS One. 2024 Dec 27;19(12):e0315320. doi: 10.1371/journal.pone.0315320 (PMC11676553; doi:10.1371/journal.pone.0315320)
Supplement: S1 File — (PDF) [file pone.0315320.s001.pdf]

# Supporting information

Below are the otolith strontium isotopic plots of all of the additional (see also Figures 3 in the main text) Northern Pike caught at Vogel Lake.

Figure S1:

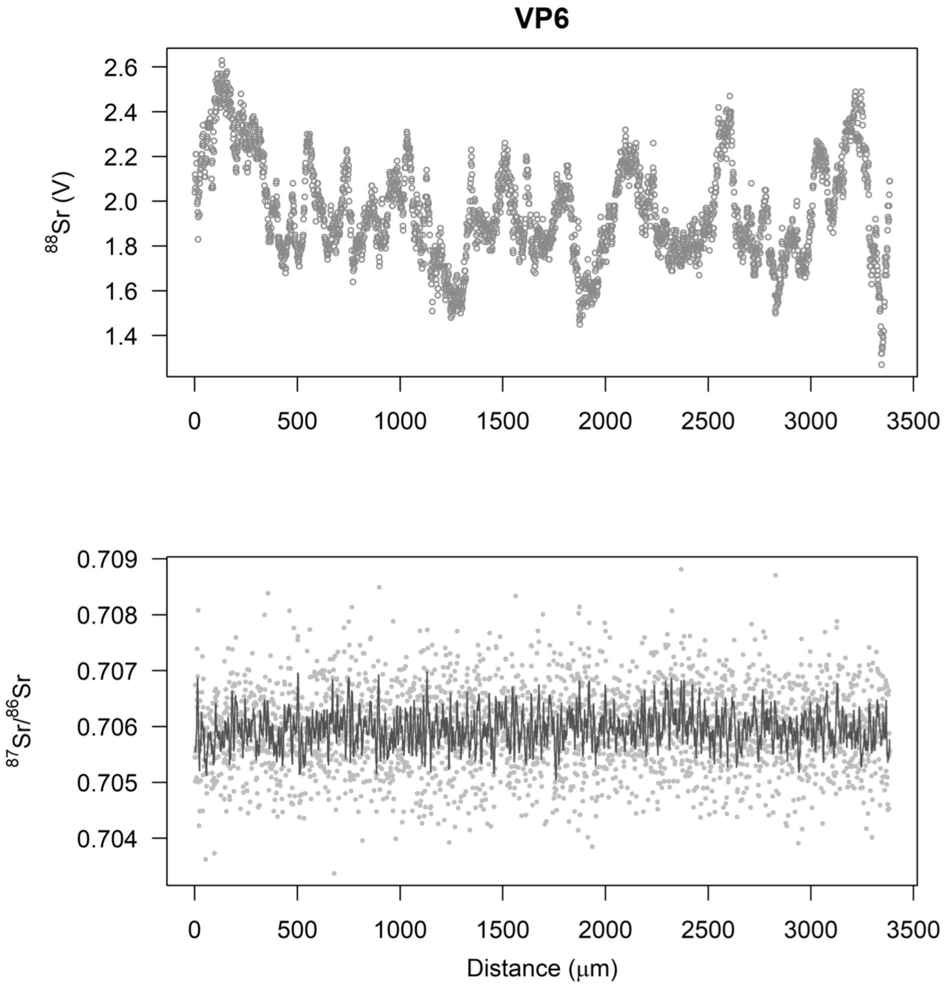

Figure S2:

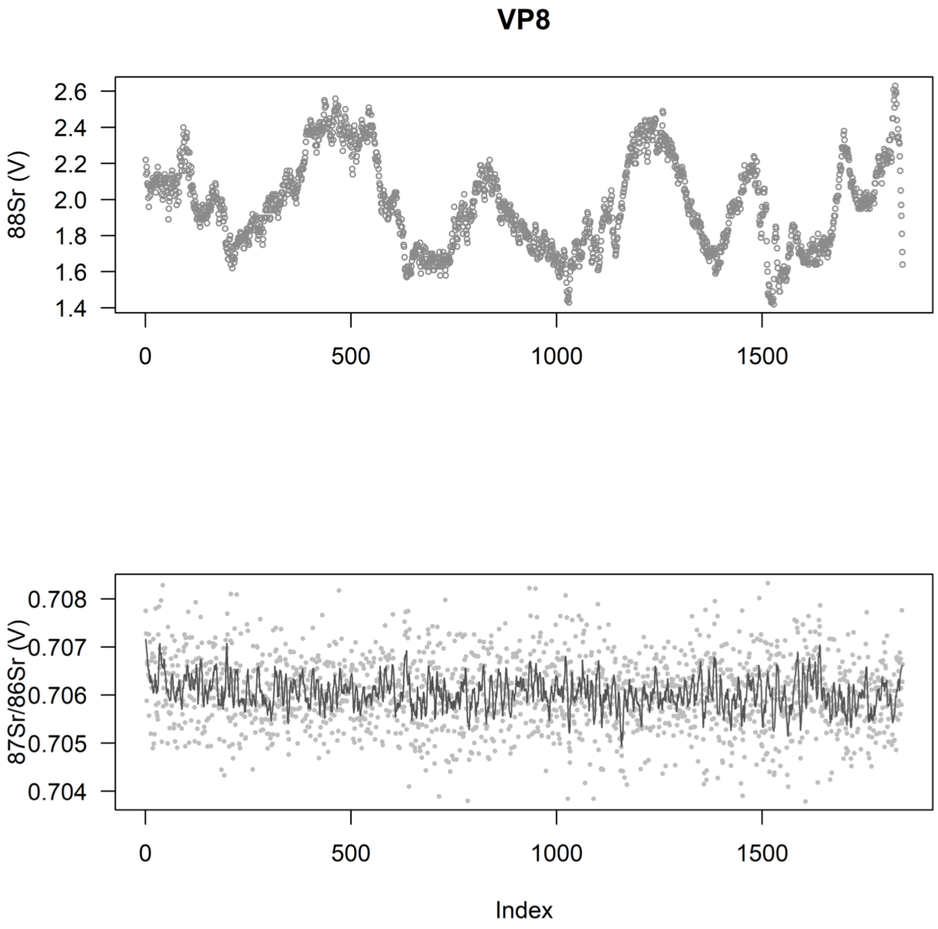
VP8
88Sr (V)
2.6
2.4
2.2
2.0
1.8
1.6
1.4
0
500
1000
1500
87Sr/86Sr (V)
0.708
0.707
0.706
0.705
0.704
0
500
1000
1500
Index

Figure S3:

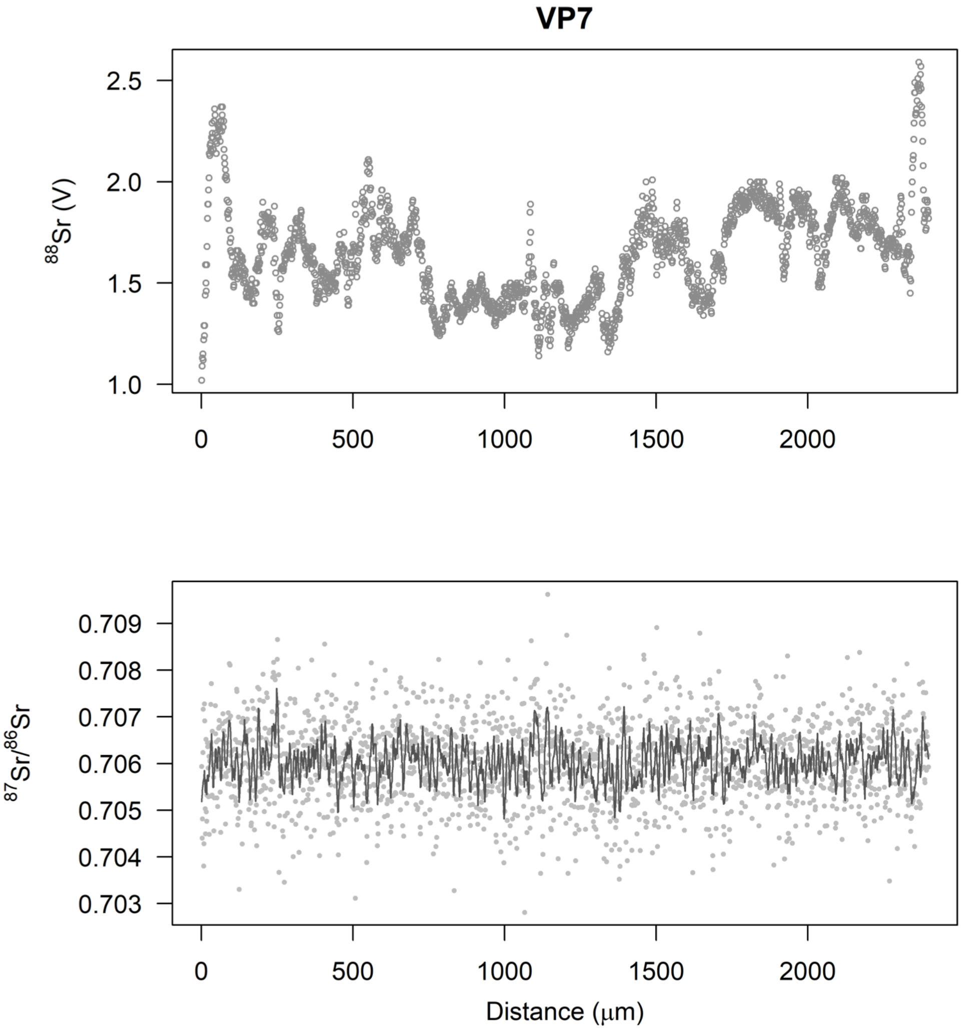

VP7
2.5
2.0
1.5
1.0
$^{88}Sr$ (V)
0
500
1000
1500
2000
0.709
0.708
0.707
0.706
0.705
0.704
0.703
$^{87}Sr/^{86}Sr$
0
500
1000
1500
2000
Distance (μm)

Figure S4:

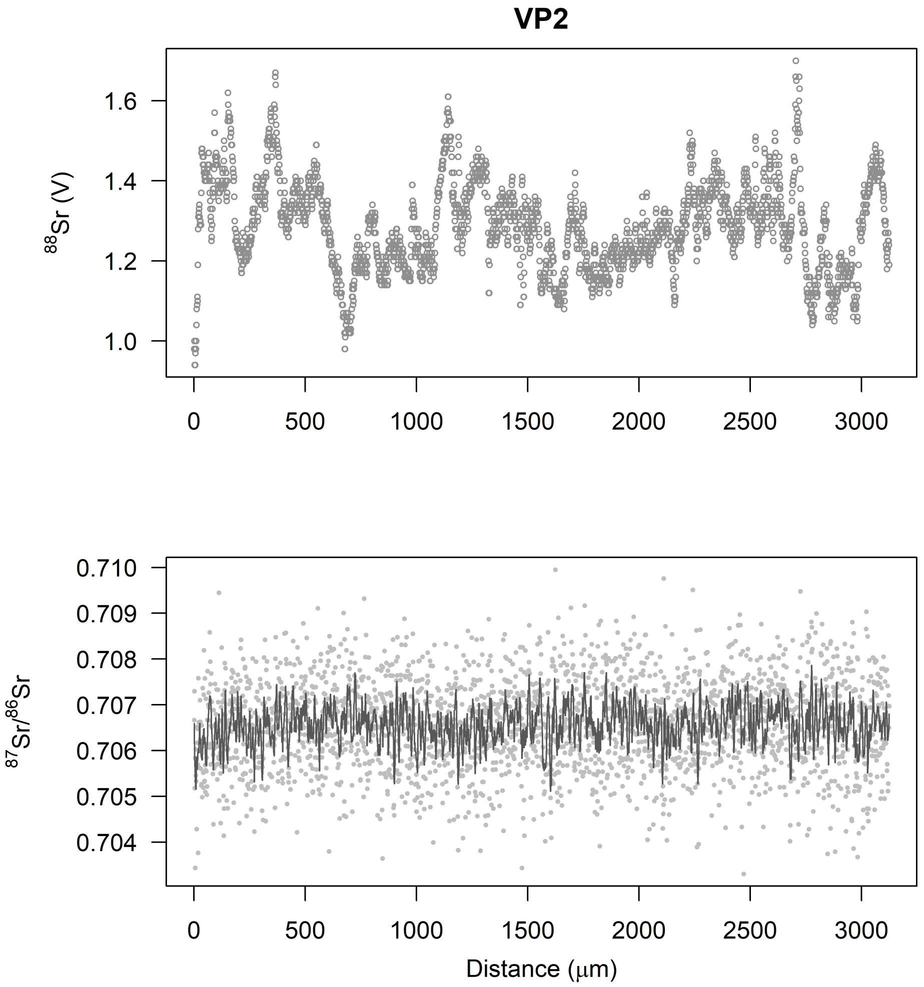

VP2
1.6
1.4
1.2
1.0
$^{88}$Sr (V)
0
500
1000
1500
2000
2500
3000
0.710
0.709
0.708
0.707
0.706
0.705
0.704
$^{87}$Sr/$^{86}$Sr
0
500
1000
1500
2000
2500
3000
Distance (μm)

Figure S5:

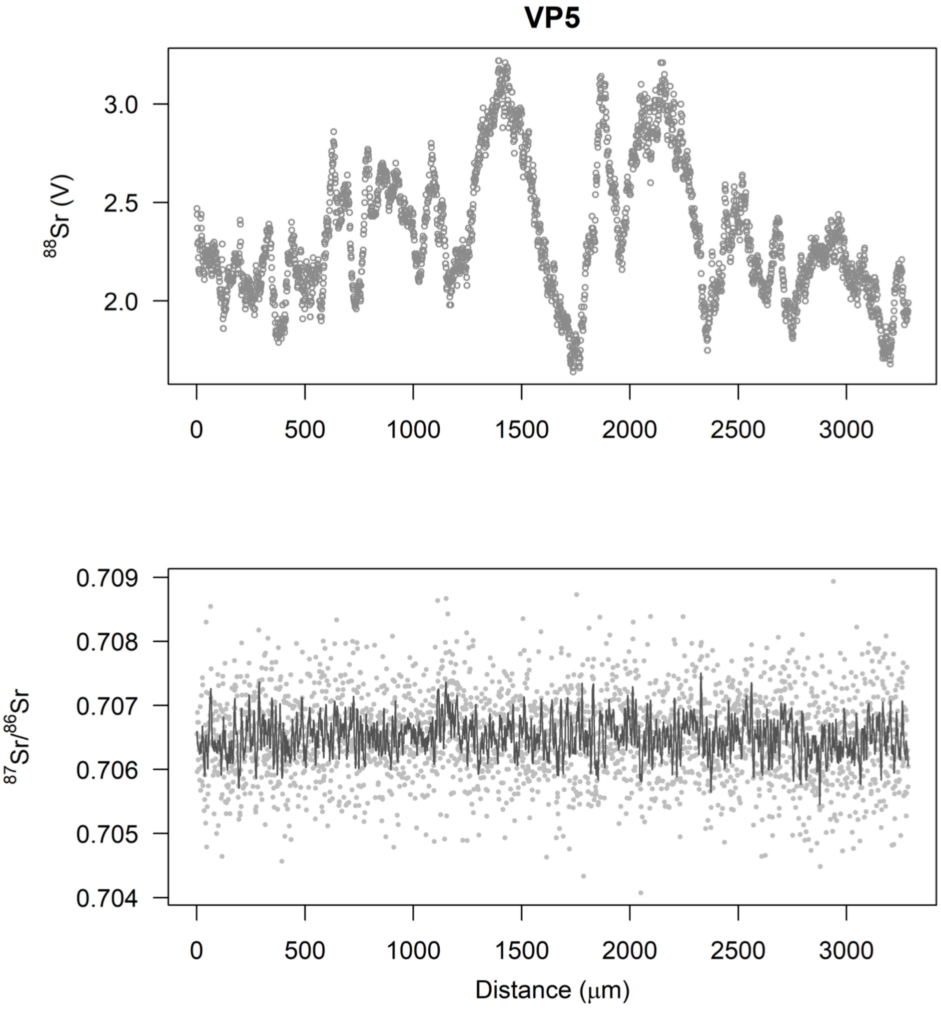
VP5
$^{88}Sr$ (V)
3.0
2.5
2.0
0
500
1000
1500
2000
2500
3000
$^{87}Sr/^{86}Sr$
0.709
0.708
0.707
0.706
0.705
0.704
0
500
1000
1500
2000
2500
3000
Distance (μm)
